# Mechanisms underlying the growth inhibitory effects of the cyclo-oxygenase-2 inhibitor celecoxib in human breast cancer cells

**DOI:** 10.1186/bcr1019

**Published:** 2005-04-04

**Authors:** Gargi D Basu, Latha B Pathangey, Teresa L Tinder, Sandra J Gendler, Pinku Mukherjee

**Affiliations:** 1 Department of Biochemistry and Molecular Biology, Mayo Clinic College of Medicine, Scottsdale, Arizona, USA

## Abstract

**Introduction:**

Inhibitors of cyclo-oxygenase (COX)-2 are being extensively studied as anticancer agents. In the present study we evaluated the mechanisms by which a highly selective COX-2 inhibitor, celecoxib, affects tumor growth of two differentially invasive human breast cancer cell lines.

**Methods:**

MDA-MB-231 (highly invasive) and MDA-MB-468 (moderately invasive) cell lines were treated with varying concentrations of celecoxib *in vitro*, and the effects of this agent on cell growth and angiogenesis were monitored by evaluating cell proliferation, apoptosis, cell cycle arrest, and vasculogenic mimicry. The *in vitro *results of MDA-MB-231 cell line were further confirmed *in vivo *in a mouse xenograft model.

**Results:**

The highly invasive MDA-MB-231 cells express higher levels of COX-2 than do the less invasive MDA-MB-468 cells. Celecoxib treatment inhibited COX-2 activity, indicated by prostaglandin E_2 _secretion, and caused significant growth arrest in both breast cancer cell lines. In the highly invasive MDA-MB-231 cells, the mechanism of celecoxib-induced growth arrest was by induction of apoptosis, associated with reduced activation of protein kinase B/Akt, and subsequent activation of caspases 3 and 7. In the less invasive MDA-MB-468 cells, growth arrest was a consequence of cell cycle arrest at the G_0_/G_1 _checkpoint. Celecoxib-induced growth inhibition was reversed by addition of exogenous prostaglandin E_2 _in MDA-MB-468 cells but not in MDA-MB-231 cells. Furthermore, MDA-MB-468 cells formed significantly fewer extracellular matrix associated microvascular channels *in vitro *than did the high COX-2 expressing MDA-MB-231 cells. Celecoxib treatment not only inhibited cell growth and vascular channel formation but also reduced vascular endothelial growth factor levels. The *in vitro *findings corroborated *in vivo *data from a mouse xenograft model in which daily administration of celecoxib significantly reduced tumor growth of MDA-MB-231 cells, which was associated with reduced vascularization and increased necrosis in the tumor mass.

**Conclusion:**

The disparate molecular mechanisms of celecoxib-induced growth inhibition in human breast cancer cells depends upon the level of COX-2 expression and the invasive potential of the cell lines examined. Data suggest a role for COX-2 not only in the growth of cancer cells but also in activating the angiogenic pathway through regulating levels of vascular endothelial growth factor.

## Introduction

The incidence of breast cancer – the second leading cause of cancer death in women in the USA – is increasing, and current therapy is unable to achieve clinical responses in patients with highly invasive metastatic disease. There is a consequent need for more effective approaches to prevention and treatment of breast cancer. Nonsteroidal anti-inflammatory drugs (NSAIDs) show great promise in this respect. Recent data on regular NSAID use for 5–9 years indicated a 21% reduction in the incidence of breast cancer, and regular NSAID use for 10 or more years produced a 28% reduction in the incidence of breast cancer [1]. Preclinical studies [2-4] have consistently shown that NSAIDs inhibit mammary carcinogenesis.

Various mechanisms may be responsible for the observed effects of NSAIDs against breast cancer. Inhibition of cyclo-oxygenase (COX), particularly the COX-2 isozyme, and blockade of the prostaglandin (PG) cascade may have impacts on neoplastic growth and development by inhibiting several key features of mammary carcinogenesis – namely proliferation, angiogenesis and metastasis. Inhibition of COX also causes induction of apoptosis in malignant cells and enhances antineoplastic activity of cytotoxic T lymphocytes [5-8]. Our study conducted in newly diagnosed stage I and stage II breast cancer patients [9] showed impaired functionality of T cells and dendritic cells, which correlated with COX-2 overexpression in the tumors and increased levels of PGE_2 _in the serum and tumor milieu. Therefore, a convincing case has been made for COX-2 being an important target for the antineoplastic action of NSAIDs. Unlike NSAIDs, COX-2 selective inhibitors such as celecoxib and rofecoxib do not inhibit COX-1 and thus show promise as drugs that spare the gastrointestinal system.

COX-2 is overexpressed in breast cancer tissues, and greater extent of its expression is associated with poorer prognosis [10]. Various environmental and nutritional risk factors induce COX-2 expression in animal models of breast cancer [11,12]. Moreover, COX-2 selective inhibitors significantly delayed the incidence of mammary tumors in transgenic mice expressing the Her2/Neu, and polyoma-middle T oncogenes [13,14]. Recently, a transgenic mouse model was developed in which the human COX-2 gene was expressed in the mammary gland under the control of the murine mammary tumor virus promoter [15]. That study demonstrated that enhanced COX-2 expression strongly predisposes to transformation of the mammary gland in multiparous animals. These data strongly suggest that local expression of COX-2 is sufficient for *in situ *tumor initiation and/or progression. Another transgenic overexpression study with COX-2 targeted to the epidermis also supports the concept that COX-2 is a critical regulator of tumor progression [16]. Transfections of the breast cancer cell line Hs578T with cDNA for COX-2 led to an increase in expression and activity of matrix metalloproteinase-2, resulting in increasingly invasive behavior of the cells [17]. COX-2 specific inhibitors have the ability to block cell growth, and induce apoptosis and cell cycle arrest in murine mammary tumor cell lines [18]. However, the molecular mechanisms involved are not well understood. If COX-2 inhibitors act only by modulating COX-2 expression, then that would imply that this therapy would be limited to COX-2 overexpressing tumors; hence, this question is of considerable clinical importance.

In the present study we established that the level of COX-2 expression and the invasive property of breast cancer cells determines the mechanism of celecoxib-induced growth inhibition; that COX-2 is involved in extracellular matrix associated microvascular channel formation by breast cancer cells; and that COX-2 inhibits angiogenesis *in vivo*. The study should further our understanding of the cellular and molecular mechanisms underlying the chemopreventive effect of a COX-2 selective inhibitor in breast cancer. To the best of our knowledge, this is the first study demonstrating the diverse mode of action of celecoxib on human breast cancer cells, which may be dependent upon the cells' invasive properties and levels of COX-2 expression. This is also the first report suggesting a direct role for COX-2 in matrix associated microvascular channel formation by breast cancer cells.

## Methods

### Cell culture

The human breast cancer cell lines MDA-MB-231 and MDA-MB-468 were obtained from the American Type Culture Collection (ATCC; Rockville, MD, USA) and cultured following instructions from the ATCC. Briefly, cells were grown in Dulbecco's modified eagle medium (DMEM; GIBCO-BRL, Rockville, MD, USA) supplemented with 5% fetal calf serum (FCS), 100 U penicillin, 0.1 μg streptomycin and 2 mmol/l L-glutamax. Cells were maintained in log phase in 37°C incubator with 10% carbon dioxide. For each experiment cells were plated in FCS-containing media in 58 cm^2 ^culture dishes at a cell density of approximately 1 × 10^6 ^cells/dish and incubated for another 48 hours. Cell cultures were treated with increasing concentrations of celecoxib (20–60 μmol/l; Pfizer, New York, NY, USA) and with dimethyl sulfoxide (DMSO; the vehicle in which celecoxib was dissolved). The concentration of celecoxib used in our experiments is clinically relevant because the serum concentrations of COX-2 inhibitors in patients range from 20 to 100 μmol/l [19]. The concentrations used in the study are based on our titrations with celecoxib for the two cell lines and from several published references on other cell lines [20-22]. In both the cell lines tested there was no evidence of apoptosis or cell cycle arrest at concentrations below 20 μmol/l.

### SDS-PAGE immunoblotting

Following harvesting of adherent cells by scraping, cell lysates were prepared and quantified by BCA assay. Lysates (100 μg) were resolved on a 10–15% acrylamide gel and electroblotted onto immobilon-P polyvinylidene diflouride membranes (Sigma, St. Louis, MO, USA). These were probed with primary antibodies for COX-2 (p66), BAX (p23), Bcl-2 (p26), and vascular endothelial growth factor (VEGF; p20), all from Santa Cruz Biotechnology Inc. (Santa Cruz, CA, USA), and phosphorylated Akt (pAkt; p60; Cell Signaling, Beverly MA, USA), and then probed with the appropriate secondary antibodies. Bound antibodies were detected using an enhanced chemiluminescence detection kit (SuperSignal West Dura, Pierce, Rockford, IL, USA), and developed on high performance chemiluminescence films (Amersham Pharmacia Biotech, Piscataway, NJ, USA).

### Proliferation assay

Cell proliferation was determined by using [^3^H]thymidine incorporation, in which 1 μCi of [^3^H]thymidine was added to the drug or vehicle treated cultures 16 hours before harvesting using a Packard Cell Harvester (Packard Biosciences, Shelton, CT, USA). Incorporated thymidine was evaluated using the Topcount micro-scintillation counter (Packard Biosciences). Results were expressed as [^3^H]thymidine uptake. All determinations were performed in triplicate. Proliferation is directly correlated to radioactive counts/min. In order to determine whether added PGE_2 _could counteract the growth inhibitory effect of celecoxib, we treated cells with celecoxib (40 μmol/l) and 12.5–200 pg/ml PGE_2 _and incubated them for 96 hours before determining [^3^H]thymidine incorporation, as mentioned above.

### Assay for apoptosis

Following treatment of cells with celecoxib for 48 hours, apoptosis was determined by staining the cells with annexin V and propidium iodide (PI), in accordance with the manufacturer's instructions for use of the BD Pharmingen (San Diego, CA, USA) apoptosis kit. Briefly, an aliquot of 10^5 ^cells was incubated with annexin V–fluorescein isothiocyanate and PI for 15 min at room temperature in the dark. Cells were immediately analyzed by flow cytometry. Viable cells exclude PI and are negative for annexin V staining, whereas early apoptotic cells are annexin V positive and PI negative. Cells that are not viable due to apoptotic cell death stain positive for annexin V and PI. The percentage of stained cells in each quadrant was quantified using CellQuest software (BD Biosciences, San Jose, CA, USA) and the total number of apoptotic cells (both early and late apoptosis) was quantified.

### Confocal microscopy for detection of apoptotic bodies

Cells were grown with celecoxib (60 μmol/l) for 48 hours and then trypsinized. Cells were resuspended in phosphate-buffered saline (PBS) with 0.1% bovine serumn albumin at a final concentration of 5 × 10^7 ^cells/ml and 2 μl of 5 mmol/l carboxyfluoroscein succinimidyl ester (CFSE)/ml (Molecular Probes, Eugene, OR, USA) was added. After 10 min of incubation at 37°C the staining was quenched by adding five times the volume of ice-cold PBS and excess stain was washed off by repeated washes in PBS. Cells were fixed in 95% ethanol for 1 hour on ice and resuspended in PBS containing 20 μg/ml PI (Sigma) and 15 μg/ml RNase A (Sigma). Images were captured on the LSM510 confocal microscope (Carl Zeiss Inc., Gottingen, Germany) using excitation wavelengths of 488 nm (for CFSE) and 543 nm (for PI).

### Assay for caspases 3 and 7

To evaluate whether celecoxib treatment can induce activation of caspases 3 and 7, we detected levels of active forms of caspases 3 and 7 in cell lysates from treated and untreated cells using the EnzChek Caspase-3/7 Assay Kit (Molecular Probes), in accordance with the manufacturer's protocol. In principle, active caspase 3 or 7 cleaves a fluorogenic substrate; this releases the fluorochrome, which is detected using a spectrofluorometer.

### Cell cycle analysis

Cells were treated with increasing concentrations (20–60 μmol/l) of celecoxib or DMSO (vehicle) in medium supplemented with 5% FCS for 48 hours. The adherent and the nonadherent cell fractions were harvested and cell pellets were fixed and permeabilized in 95% cold ethanol, and resuspended in PBS containing 20 μg/ml PI (Sigma) and 15 μg/ml RNase A (Sigma). Samples were incubated in the dark at 37°C for 30 min and analyzed by flow cytometry (Becton Dickinson, San Diego, CA, USA). For each sample, 50,000 fluorescent cells were counted. Data were analyzed using the ModFit software (Verity Software House Inc., Topsham, ME, USA) to determine DNA content and cell cycle phase (G_0_/G_1_–S–G_2_/M phase). Cell doublets and clumps were eliminated from the analyses by gating.

### Prostaglandin E_2 _production

Cells were treated with increasing concentrations (20–60 μmol/l) celecoxib or DMSO (vehicle) in medium supplemented with 5% FCS for 48 hours. Levels of PGE_2 _released in media were measured using a PGE_2 _enzyme immunoassay kit from Cayman Chemical Co. (Ann Arbor, MI, USA). Medium was sampled, centrifuged to remove floating cells and frozen immediately at -70°C until assay. The PGE_2 _assay was performed in accordance with the manufacturer's instructions, following dilution to ensure that readings were within the limits of accurate detection by the assay. The results are expressed as pg PGE_2_/ml ± standard deviation.

### Assay for vasculogenic mimicry

This assay was performed as described [23]. Cells were grown until they were about 80% confluent. The growth medium was replaced with serum-free DMEM supplemented with 100 μg/ml heparin (Elkins-Sinn, Inc. Cherry Hill, NJ, USA) and antibiotics, and cells were incubated for 24 additional hours. The cells were trypsinized, counted, and resuspended in media (at a concentration of 4 × 10^4 ^cells/ml) containing 40 and 60 μmol/l concentrations of celecoxib or vehicle. The wells of a 24-well tissue culture plate were evenly coated with 0.1 ml/well growth factor reduced Matrigel (BD Biosciences), which was allowed to solidify at 37°C for 30 min, in accordance with the manufacturer's instructions, before the cells were plated. The cell suspension was plated (1 ml/well) onto the surface of Matrigel and incubated at 37°C for 48 hours and photographed using a Nikon inverted phase contrast photomicroscope (Nikon USA, Garden City, NY, USA). Channel formation was quantified as percentage of channels formed by counting the number of connected cells in five randomly selected fields, using 200× magnification, and dividing the number by the total number of cells in the same field.

### Xenografts

Male athymic nude mice (age 6–8 weeks) were obtained from NxGen Biosciences Inc. (San Diego, CA, USA) and animals were housed under specific pathogen-free conditions. Five mice/group were prophylactically treated with either celecoxib (25 mg/kg body weight) or vehicle DMSO for 7 days before the tumor cells were inoculated. MDA-MB-231 cells were harvested by centrifugation and 5 × 10^6 ^cells were suspended in 150 μl of serum free DMEM with an equal volume of cold liquid Matrigel (10 mg/ml). The suspension was injected subcutaneously in the mice. In order to determine the optimal cell number to be injected, titration with varying cell numbers was done on nude mice and the tumorigenicity of the cell line determined. The growth of these tumors was monitored by weekly examination, and growth rates were determined using caliper measurements. Tumor weight was calculated according to the following equation [24]: tumor weight (g) = (length (cm) × width (cm)^2^) × 0.5. Experiments were terminated 45 days after tumor cell injection. It was necessary to kill some of the mice earlier because of the aggressive nature of the tumor.

### Histologic studies

All solid tumors resulting were excised and fixed in formaldehyde, and paraffin-embedded blocks was sectioned at a thickness of 7 μm. Histologic evaluation of vascularity was determined by Masson's trichrome staining [25]. This method stains fibrous tissue and stroma green. Blood vessels containing red blood cells stain bright red. Immunohistochemical localization of factor VIII related antigen on endothelial cells was determined using the polyclonal rabbit antihuman von Willebrand factor purchased from Dako Cytomation (Glostrup, Denmark), using the manufacturer's recommended staining protocol.

### Statistical analysis

The celecoxib experiments were run in triplicate; the mean as well as standard deviations were computed. The means were then compared using one-way analysis of variance with Dunnett adjustment.

## Results

### Cyclo-oxygenase-2 protein is differentially expressed in breast cancer cell lines

**Figure 1:**
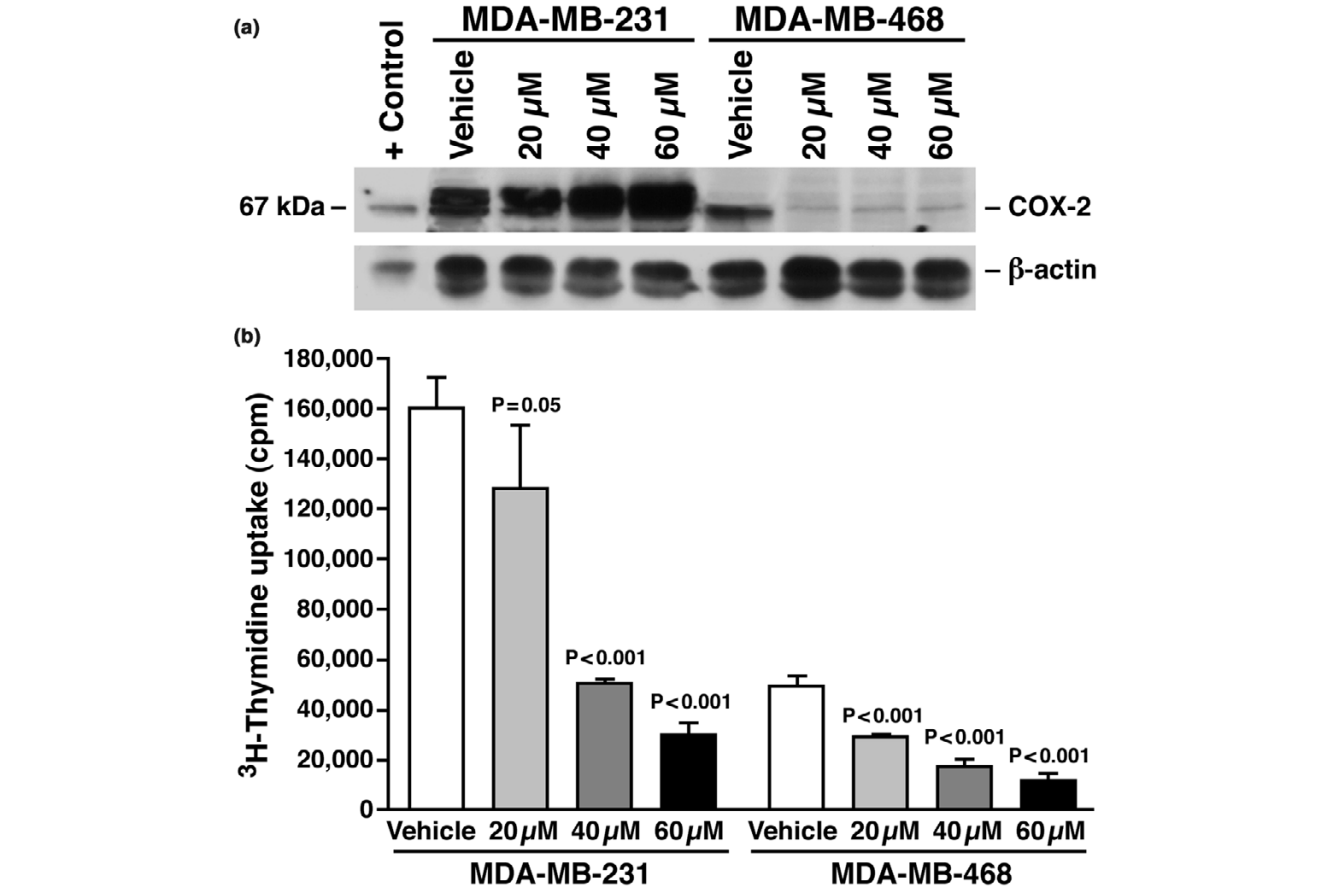
Celecoxib regulates COX-2 levels and causes growth arrest in human breast cancer cells. **(a) **Cyclo-oxygenase (COX)-2 is expressed in both MDA-MB-231 and MDA-MB-468 cell lines. Western blot analysis of vehicle and celecoxib (20–60 μmol/l) treated cells. SDS-PAGE electrophoresis was performed using a 10% resolving gel. Protein was loaded at 100 μg per lane. Lipopolysaccharide/phorbol 12-myristate 13-acetate treated whole cell lysate from RAW264.7 cell line was used as positive control. Gels were blotted and probed with COX-2 monoclonal antibody. Both cell lines expressed COX-2. MDA-MB-231 cells expressed higher levels of COX-2 than did MDA-MB-468 cells. With drug treatment, COX-2 protein level did not change in the MDA-MB-231 cells, but there was reduction in the level of COX-2 protein in the MDA-MB-468 cells after treatment. β-Actin blot is included to confirm equal loading. These experiments were repeated three times with similar results. **(b) **Celecoxib induced dose-dependent inhibition of proliferation of breast cancer cell lines. Cells were incubated for 4 days with vehicle or celecoxib, and [^3^H]thymidine was added 24 hours before harvest. After washing off excess thymidine, cells were lysed with 5% Triton X-100, and incorporated thymidine was evaluated. Celecoxib treatment caused significant dose-dependent growth inhibition in both human breast cancer cell lines. Mean values of three experiments ± standard deviation is shown. *P *values represent significant differences between vehicle control and celecoxib treatment.

We studied two human breast cancer cell lines, MDA-MB-231 and MDA-MB-468, for COX-2 expression by western blotting. Both cell lines expressed COX-2, although MDA-MB-468 cells exhibited lower protein expression than did MDA-MB-231 cells. Western blot analysis for COX-2 protein in the MDA-MB-231 cell line showed little change in protein expression after treatment with 20–40 μmol/l celecoxib. At the level of 60 μmol/l there was a slight increase in COX-2 expression. However, in the MDA-MB-468 cell line there was significant downregulation of COX-2 expression upon drug treatment (Fig. 1a).

### Celecoxib inhibits growth and proliferation of breast cancer cell lines

Celecoxib at concentrations of 20, 40, and 60 μmol/l was used to treat the two cell lines for 48 hours. Under the phase contrast microscope, both cell lines exhibited a dramatic morphologic change as well as growth arrest after 48 hours of drug treatment (data not shown). The rate of proliferation in response to celecoxib treatment was assayed by measuring incorporation of [^3^H]thymidine uptake. Significant inhibition of proliferation was observed in both cell lines in a dose-dependent manner, in response to varying concentrations of celecoxib at 96 hours after treatment (*P *< 0.001; Fig. 1b). Similar growth inhibition was observed at earlier time points (48 and 72 hours after treatment (data not shown).

### Celecoxib induces apoptosis in MDA-MB-231 but not in MDA-MB-468 cells

**Figure 2:**
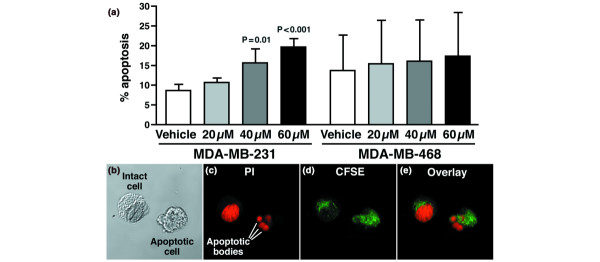
Celecoxib induces apoptosis in MDA-MB-231 cells. **(a) **Flow cytometric analysis of vehicle-treated and celecoxib-treated cells stained with annexin V and propidium iodide (PI) was done 48 hours after treatment. The population shown in the figure is total apoptotic cells, which includes early and late apoptosis. Significant induction of apoptosis was observed in the MDA-MB-231 cells at 40 and 60 μmol/l concentrations of celecoxib. Apoptosis was not induced in MDA-MB-468 cells. Mean values of three experiments ± standard deviation is shown. *P *values represent significant differences between vehicle control and celecoxib treatment. **(b–e) **Celecoxib induces formation of apoptotic bodies in MDA-MB-231 cells. Shown are confocal images of MDA-MB-231 cells subjected to 48 hours of celecoxib (60 μmol/l) treatment. Cells were stained with CFSE (panel d) and then fixed in 95% ethanol and stained with PI (panel c). Cells were visualized in a confocal microscope (Carl Zeiss Inc.) using excitation wavelengths of 488 nm (for CFSE) and 543 nm (for PI). Loss of integrity of nuclear envelope and formation of peripheral, sharply delineated masses of condensed chromatin or apoptotic bodies are visualized. Panel b represents phase contrast images of the cells and panel e represents colocalization of CFSE and PI. Images were taken 200×.

Because COX inhibitors have been reported to mediate apoptosis in many cells [26,27], we investigated whether the observed growth inhibition mediated by celecoxib was associated with induction of programmed cell death. Flow cytometric analysis of annexin V/PI staining in celecoxib-treated and vehicle-treated cells was used to analyze apoptosis. Following 48 hours of drug treatment, induction of apoptosis was observed in the MDA-MB-231 cells in a dose-dependent manner (Fig. 2a). Celecoxib at concentrations of 40 and 60 μmol/l caused significant increases in the percentage apoptotic cells (*P *= 0.01 and *P *< 0.001, respectively). In the MDA-MB-468 cell line apoptosis was not induced with celecoxib treatment (Fig. 2a). In spite of the lack of evidence of increased apoptosis, MDA-MB-468 cells had significantly lower proliferation after drug treatment (Fig. 1b). Treated cells appeared rounded up and exhibited atypical morphology (data not shown), which suggested that alterations in the adhesive properties of these cells might have occurred and other pathways may be involved in the growth inhibition observed in MDA-MB-468 cells.

### Celecoxib induces formation of apoptotic bodies and loss of nuclear envelope integrity in MDA-MB-231 cells

To follow up on the celecoxib-induced apoptosis of the MDA-MB-231 cells, we analyzed morphological changes in MDA-MB-231 cells after celecoxib treatment using confocal microscopy. Celecoxib at concentrations of 40 and 60 μmol/l caused loss of integrity of nuclear envelope and induced formation of peripheral, sharply delineated masses of condensed chromatin or apoptotic bodies, which are characteristic structural features of apoptosis (Fig. 2b–e). Membrane blebbing was also observed, along with loss of plasma membrane integrity in some cells (data not shown). These results indicate that celecoxib treatment caused architectural changes in membrane and cell nucleus within 48 hours of treatment. No such changes were observed in MDA-MB-468 cells (data not shown), which correlated with our observation that there was no significant induction of apoptosis in these cells after celecoxib treatment (Fig. 2a).

### Celecoxib inhibits activation of protein kinase B/Akt kinase in MDA-MB-231 cells

**Figure 3:**
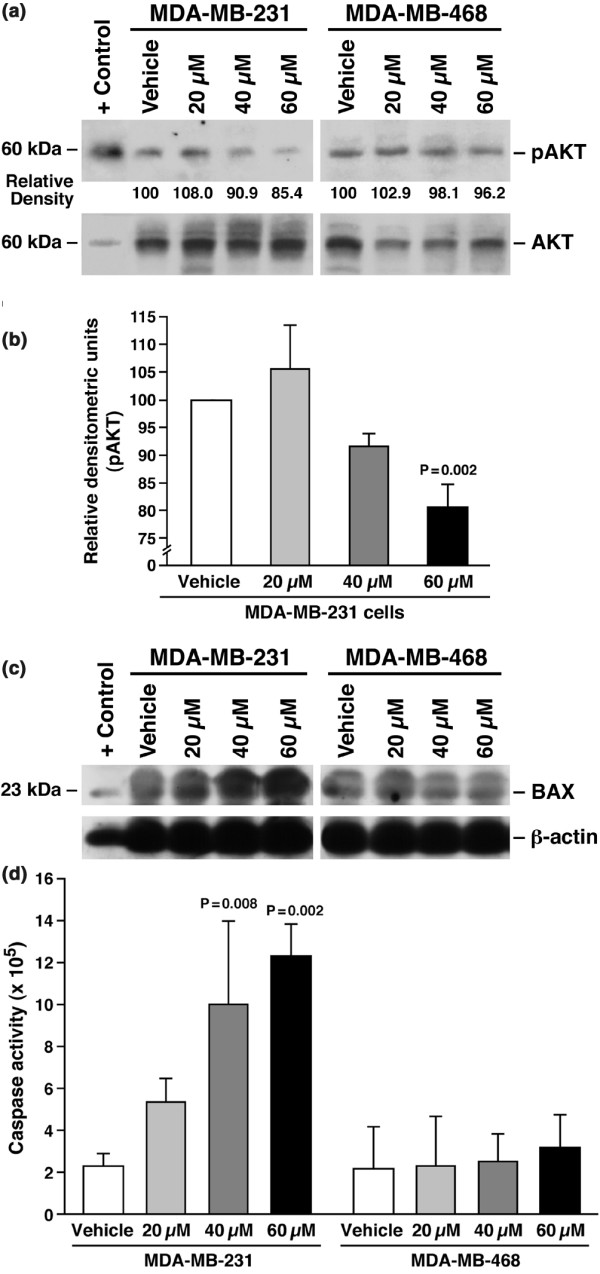
Celecoxib induced down-regulation of pAkt, increase in Bax, and caspase 3/7 in MDA-MB-231 cells. (a) Total Akt and phosphorylated Akt (pAkt). Western blot analysis of cell lysates prepared from vehicle and celecoxib (20–60 μmol/l) treated cells. SDS-PAGE electrophoresis was performed using 10% resolving gel. Protein was loaded at 100 μg per lane and the protein of interest was detected using specific antibodies. Celecoxib treatment at 40 and 60 μmol/l caused decreases in the levels of pAkt in MDA-MB-231 cells, with no change in MDA-MB-468 cells. Numbers below each lane represents percentage of protein expression compared with vehicle-treated cell lysate, which was set to equivalent to 100%, as determined by densitometric analysis. Control cell extracts from Jurkats were used as positive control for Akt and pAkt. **(b) **Average densitometric values of three separate experiments showing reduction in pAkt with celecoxib treatment. There was a significant decrease (*P *= 0.002) in the levels of pAkt with 60 μmol/l celecoxib treatment. **(c) **Western blot analysis of BAX. Increased expression of BAX protein was observed with increasing concentrations of celecoxib in MDA-MB-231 cells but not in MDA-MB-468 cells. The experiment was repeated three times with similar results. A β-actin blot is included to show equal loading. **(d) **Spectrofluorometric analysis of lysates prepared from vehicle and celecoxib (20–60 μmol/l) treated cells at 48 hours. Activity of caspases 3 and 7 was monitored by enzymatic cleavage using a fluorescence microplate reader with excitation at 485 ± 10 nm and emission detection at 530 ± 12.5 nm. In MDA-MB-231 cells, activities of caspases 3 and 7 were increased significantly at 40 μmol/l and 60 μmol/l drug concentrations. No increase in caspase activity was evident in the MDA-MB-468 cells. Mean values from three experiments ± standard deviation is shown. *P *values represent significant differences between vehicle control and celecoxib treatment.

Protein kinase B, Akt, is a serine/threonine protein kinase that is involved in promoting cell survival signals through the phosphoinositide 3-kinase pathway, leading to inactivation of a series of proapoptotic proteins. Akt also represents a key signaling component in cell survival by mediating the activation of downstream effectors such as BAD [28,29] and procaspase-9 [30]. Celecoxib was recently shown to induce apoptosis of cancer cells by blocking Akt activation in rat cholangiocarcinoma and human prostate cancer cells *in vitro *[21,22]. To explore whether inhibition of Akt activation may be the mechanism responsible for induction of apoptosis in MDA-MB-231 cells, we determined the effect of celecoxib on phosphorylation of Akt on breast cancer cell lines. Breast cancer cells were exposed to varying doses of celecoxib for 48 hours, and Akt and pAkt in cell lysates were determined by western blot analysis. At a concentration of 20 μmol/l, celecoxib caused slight increase in pAkt in MDA-MB-231 cells. At a concentration of 60 μmol/l, celecoxib treatment significantly (*P *= 0.002) downregulated the level of phosphorylation of Akt in MDA-MB-231 cells but not in MDA-MB-468 cells (Fig. 3a,b), suggesting that the mechanism of apoptosis induction in MDA-MB-231 cells was, in part, dependent upon decreased phosphorylation of Akt protein. Because Akt represents a key signaling component in cell survival by activating downstream apoptotic proteins [28-31], we evaluated the levels of Bax and Bcl-2 by western blot analysis of lysates derived from both cell lines after celecoxib treatment. Treatment with celecoxib at concentrations of 40 and 60 μmol/l induced increased expression of Bax in the MDA-MB-231 cells (Fig. 3c), but no significant decrease in Bcl-2 was observed (data not shown). In MDA-MB-468 cells, in which apoptosis was not evident, levels of pAkt and Bax remained unchanged with treatment (Fig. 3a,b).

### Celecoxib induces caspase-3/7 activation in MDA-MB-231 cells

Caspases are responsible for many of the biochemical and morphological changes that occur during apoptosis. Most apoptotic signals induce intracellular cleavage of caspases 3 and 7 from an inactive precursor (p32–p35) to the active forms (p17 and p12); hence, these proteins are the most extensively studied apoptotic proteins. The effector caspases 3 and 7 proteolytically cleave and activate several other caspases as well as several other apoptotic proteins, including the DNA fragmentation protein poly-ADP-ribose polymerase (PARP), which is one of the primary activators of DNA fragmentation and cell death [32-34].

We investigated whether celecoxib induced the activation of caspase 3 and caspase 7 in MDA-MB-231 cells in which apoptosis was induced. Caspase activity is presented as fluorescence emission, which is directly proportional to activities of caspases 3 and 7. Treatment with celecoxib (40 and 60 μmol/l) for 48 hours caused significant increases in activation of caspases 3 and 7 (fivefold increase at the 40 μmol/l concentration [*P *= 0.008] and sixfold increase at the 60 μmol/l concentration [*P *= 0.002]; Fig. 3d). Caspase activation was completely blocked by incubation with the caspase inhibitor Ac-DEVD-CHO (data not shown). These results suggest that celecoxib-induced apoptosis in MDA-MB-231 cells is due to activation of caspases 3 and 7, which is corroborated by studies indicating that the blockade or absence of caspase activation is sufficient to inhibit effective apoptosis [35]. In contrast, caspase activation was not observed in celecoxib-treated MDA-MB-468 cells, which correlated with no significant increase in apoptosis with celecoxib treatment (Fig. 2).

### Celecoxib induces cell cycle arrest at the G_0_/G_1 _checkpoint in MDA-MB-468 but not in MDA-MB-231 cells

**Figure 4:**
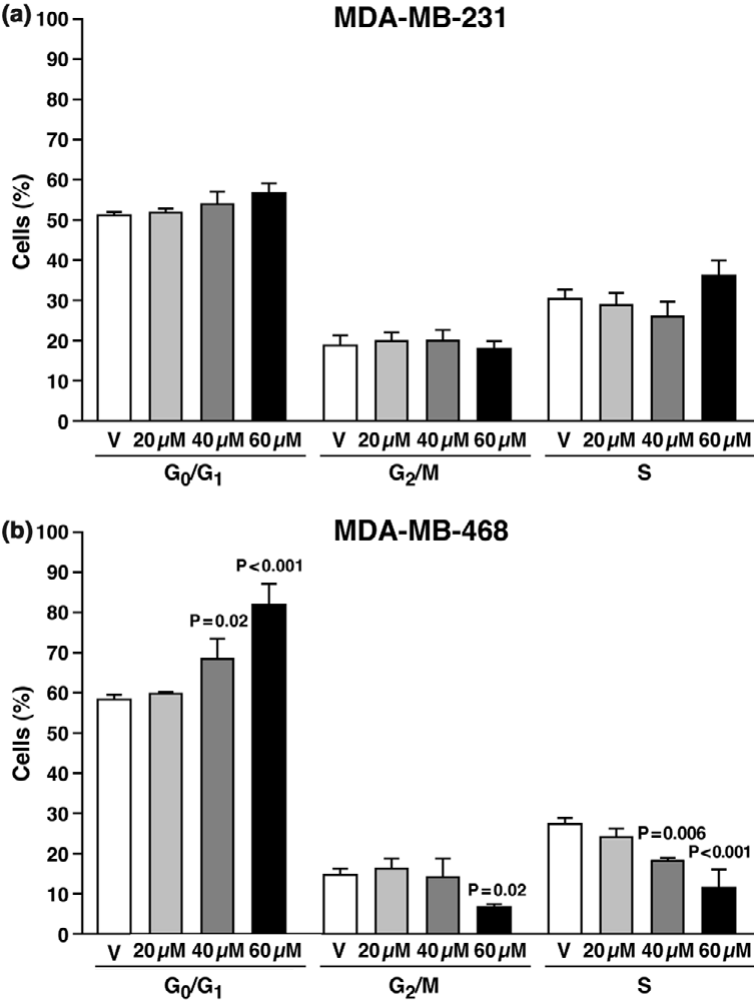
Celecoxib causes cell cycle arrest in MDA-MB-468 cells. **(a,b) **Flow cytometric analysis of cells subjected to treatment with vehicle or celecoxib (20–60 μmol/l) for 48 hours. Cells were fixed and permeabilized with 95% ethanol, stained with propidium iodide, and analyzed by flow cytometry. Celecoxib induced growth arrest at the G_0_/G_1 _cell cycle checkpoint in MDA-MB-468 cells (panel b) with no cell cycle arrest in the MDA-MB-231 cells (panel a). Mean values for three experiments ± standard deviation of the mean is shown. *P *values represent significant difference between vehicle control and celecoxib treatment. Experiments were repeated three times, with similar results.

To determine whether celecoxib-induced growth inhibition was due to changes in cell cycle progression, flow cytometric analysis was performed on cells treated with increasing concentrations of celecoxib (20–60 μmol/l) for 48 hours. In MDA-MB-468 cells, in which celecoxib did not induce apoptosis, there was induction of cell cycle arrest. At 40 and 60 μmol/l concentrations of celecoxib, significant increases (*P *= 0.02 and *P *< 0.001, respectively) in the proportion of cells that were arrested at the G_0_/G_1 _checkpoint of the cell cycle were observed. Subsequently, significant inhibition of transition to the G_2_/M phase (*P *= 0.02 at 60 μmol/l) and S phase (*P *= 0.006 and *P *< 0.001 at 40 and 60 μmol/l) was observed (Fig. 4b). Thus, growth inhibitory activity of celecoxib on these MDA-MB-468 cells was due to cell cycle arrest at G_0_/G_1 _phase and not due to induction of apoptosis. The cell cycle arrest persisted at 72 hours after drug treatment (data not shown). In MDA-MB-231 cells there was no significant difference in cell cycle progression with celecoxib treatment for 48 hours (Fig. 4a).

### Celecoxib inhibits cyclo-oxygenase-2 induced prostaglandin E_2 _production in both cell lines

COX-2 converts arachidonic acid to bioactive prostanoids. It has been demonstrated that COX-2 derived PGE_2 _is the major prostaglandin produced by breast cancer cells [36]. To determine whether COX-2 activity was affected by celecoxib treatment, PGE_2 _production using a PGE_2_-specific enzyme-linked immunosorbent assay was measured in conditioned medium collected from the breast cancer cell lines after celecoxib-treatment (20–60 μmol/l) for 48 hours. All doses of celecoxib significantly reduced PGE_2 _secretion by both cell lines (*P *< 0.01 for MDA-MB-231 and *P *= 0.03, 0.02 and 0.01 for MDA-MB-468 cells; Table 1), indicating that celecoxib is a potent inhibitor of COX-2 induced PGE_2 _production.

### Celecoxib-induced growth inhibition is reversed by exogenous prostaglandin E_2 _only in MDA-MB-468 cells

**Figure 5:**
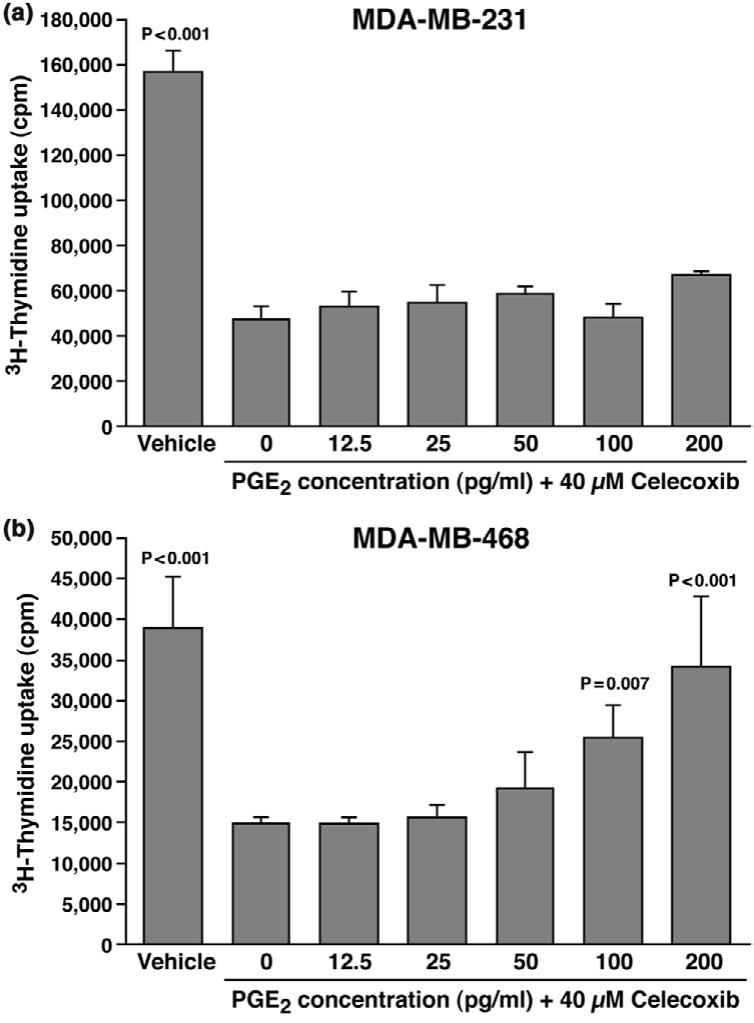
Growth inhibition of MDA-MB-468 cells was abrogated by exogenous prostaglandin (PG)E_2 _addition. [^3^H]thymidine uptake assay was done to determine proliferation of **(a) **MDA-MB-231 and **(b) **MDA-MB-468 cells treated with 40 μmol/l celecoxib with or without varying amounts of exogenous PGE_2 _(12.5–200 pg/ml). Cells were harvested after 96 hours in culture. In MDA-MB-231 cells, growth inhibition induced by 40 μmol/l celecoxib could not be restored by addition of exogenous PGE_2_; however, addition of 200 pg/ml PGE_2 _completely reversed the growth inhibition induced by 40 μmol/l celecoxib in the less invasive MDA-MB-468 cells. Average values of three experiments ± standard deviation is shown. *P *values represent significant differences between vehicle control and celecoxib treatment.

Because celecoxib caused growth inhibition in the two breast cancer cell lines and inhibited PGE_2 _secretion, we hypothesized that this growth inhibition was PGE_2 _dependent. To determine whether celecoxib-induced growth inhibition could be reversed by exogenous PGE_2_, PGE_2 _was added to cultures of MDA-MB-231 and MDA-MB-468 cells treated with constant dose (40 μmol/l) of celecoxib. Varying amounts of PGE_2 _(12.5–200 pg/ml) were added to the medium in order to take into account the fact that some of the PGE_2 _may degrade or be internalized into cells. In MDA-MB-231 cells, growth inhibition induced by 40 μmol/l celecoxib could not be restored by addition of exogenous PGE_2 _(Fig. 5a), thereby suggesting that celecoxib-induced growth inhibition in MDA-MB-231 cells may be independent of PGE_2 _levels. However, addition of 200 pg/ml PGE_2 _completely reversed the growth inhibition induced by 40 μmol/l celecoxib in the less invasive MDA-MB-468 cells (Fig. 5b), suggesting that celecoxib-induced growth regulation of these cell lines may be dependent on the levels of PGE_2_.

### Celecoxib inhibits *in vitro *matrix-associated vascular channel formation

Recent findings demonstrate the unusual ability of aggressive human breast cancer cells to form tubular structures in three-dimensional Matrigel cultures. The generation of these channels by epithelial tumor cells is called vascular mimicry [37-39]. One study [40] suggested a connection between angiogenesis and formation of these channels. Because celecoxib is known to act as an inhibitor of angiogenesis, we investigated the ability of MDA-MB-231 and MDA-MB-468 cells to form the microvascular channels with and without celecoxib treatment. MDA-MB-231 cells, which express elevated levels of COX-2 and are highly invasive, begin to form tubular structures in under 16 hours when plated on Matrigel (data not shown) and form very characterized microvascular channels by 48 hours. In contrast, MDA-MB-468 cells, which have lower COX-2 and are less invasive, start tubule formation much later, at approximately 30 hours, and exhibit significantly fewer microvascular channels at 48 hours than do MDA-MB-231 cells. These observations were specific for the high or moderately invasive cells, because the noninvasive breast cancer cells (ZR-75-1) did not form channels *in vitro *under identical culture conditions (data not shown).

**Figure 6:**
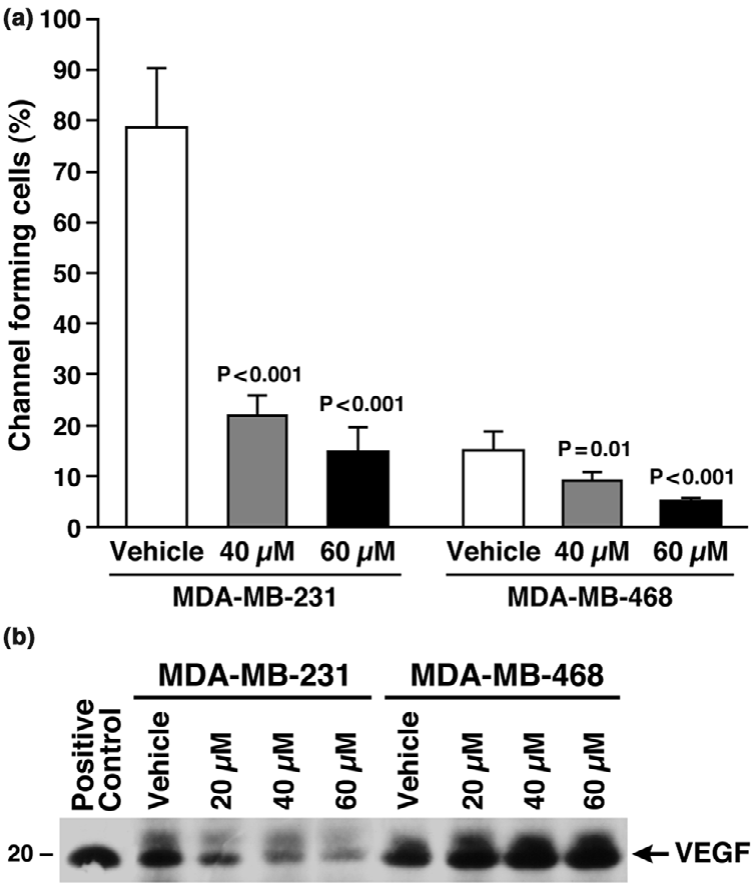
Celecoxib treatment causes reduction in microvascular channel formation by regulating VEGF levels. **(a) **The percentage of cells forming channels was much greater in MDA-MB-231 cells than in MDA-MB-468 cells. In both cells, treatment with 40 and 60 μmol/l celecoxib caused significant reduction in the number of channels. *P *values represent significant differences between vehicle control and celecoxib treatment. **(b) **Western blot analysis of cell lysates prepared from vehicle and celecoxib (20–60 μmol/l) treated cells. SDS-PAGE electrophoresis was performed using 15% resolving gel. Protein was loaded at 100 μg per lane and the protein was detected using vascular endothelial growth factor (VEGF) antibody. Celecoxib treatment decreased VEGF levels in MDA-MB-231 cells in a dose-dependent manner.

We found that celecoxib treatment at concentrations of 40 and 60 μmol/l was able to reduce significantly the formation of channels in both breast cancer cell lines in a dose-dependent manner, as compared with vehicle treated cells (*P *< 0.001 for MDA-MB-231 cells and *P *< 0.001 for MDA-MB-468 cells; Fig. 6a), suggesting a role for COX-2 in channel formation. The effect of celecoxib on channel formation was only quantified on live adherent cells in Matrigel as the apoptosed and dead cells float into the media. Thus, we believe that the negative effect of celecoxib on channel formation was not due to cell death, which was also measured by trypan blue exclusion (data not shown).

### Celecoxib inhibits expression of vascular endothelial growth factor protein in MDA-MB-231 cells

Recent reports have shown that a nonspecific COX inhibitor (indomethacin) suppresses the expression of VEGF gene expression *in vitro *in mammary tumor cells [41]. We evaluated the levels of VEGF protein from tumor lysate of cells treated with vehicle or increasing doses of celecoxib. Compared with control, celecoxib (20–60 μmol/l) treatment reduced expression of VEGF in the MDA-MB-231 cells in a dose-dependent manner (Fig. 6b). No such reduction was observed in the MDA-MB-468 cells treated with celecoxib (Fig. 6b), suggesting that in the highly aggressive MDA-MB-231 cells the COX-2/PGE_2 _pathway may play a critical role in channel formation and angiogenesis, in part by activating proangiogenic proteins such as VEGF. Future studies will evaluate other proteins associated with the angiogenic pathway.

### *In vivo *tumor growth was reduced with celecoxib treatment

**Figure 7:**
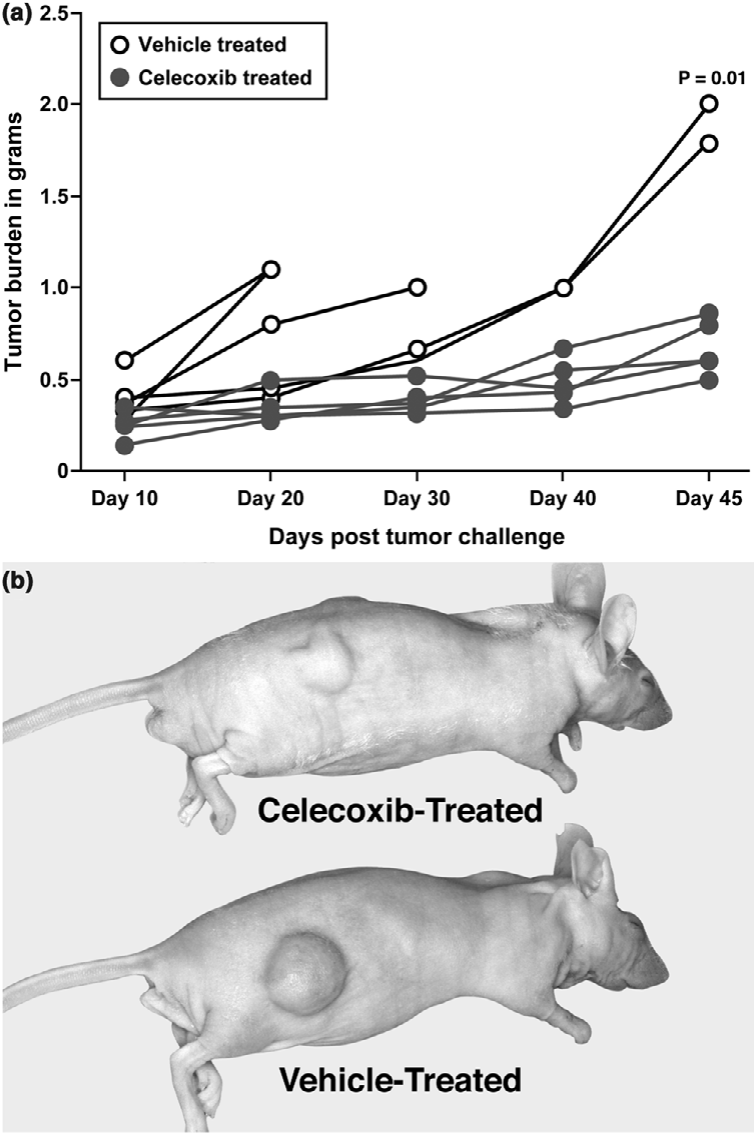
Celecoxib treatment reduced MDA-MB-231 tumor growth in nude mice. **(a) **Five mice per group were treated with either celecoxib (25 mg/kg body weight) or vehicle (dimethyl sulfoxide) and the mice were killed 45 days after the tumor cells were inoculated. Tumor growth was monitored by weekly examination using digital calipers, and tumor weight was calculated using to the following equation [23]: tumor weight (g) = (length (cm) × width (cm)^2^) × 0.5. Three mice from the vehicle-treated group had to be killed early because of the aggressive nature of the tumor. The other two mice in the vehicle-treated group had significantly greater tumor burden (*P *= 0.01) than did the five mice in celecoxib-treated group. **(b) **A representative mouse from each treatment group is illustrated; lower tumor mass Is evident in the treated animal as compared with the vehicle control.

Nude mice were prophylactically treated with celecoxib or vehicle for 1 week before tumor challenge with MDA-MB-231 cells in Matrigel. Celecoxib treatment was continued for 45 days after tumor challenge. Mice treated with celecoxib (25 mg/kg body weight) exhibited significant (*P *= 0.01) reduction in tumor growth as compared with vehicle-treated mice without evidence of systemic toxicity (Fig. 7a). A representative mouse from each treatment group is shown in Fig. 7b; the treated mouse has reduced tumor mass compared with the control mouse.

### *In vivo *inhibition of angiogenesis and increase in necrosis with celecoxib treatment

**Figure 8:**
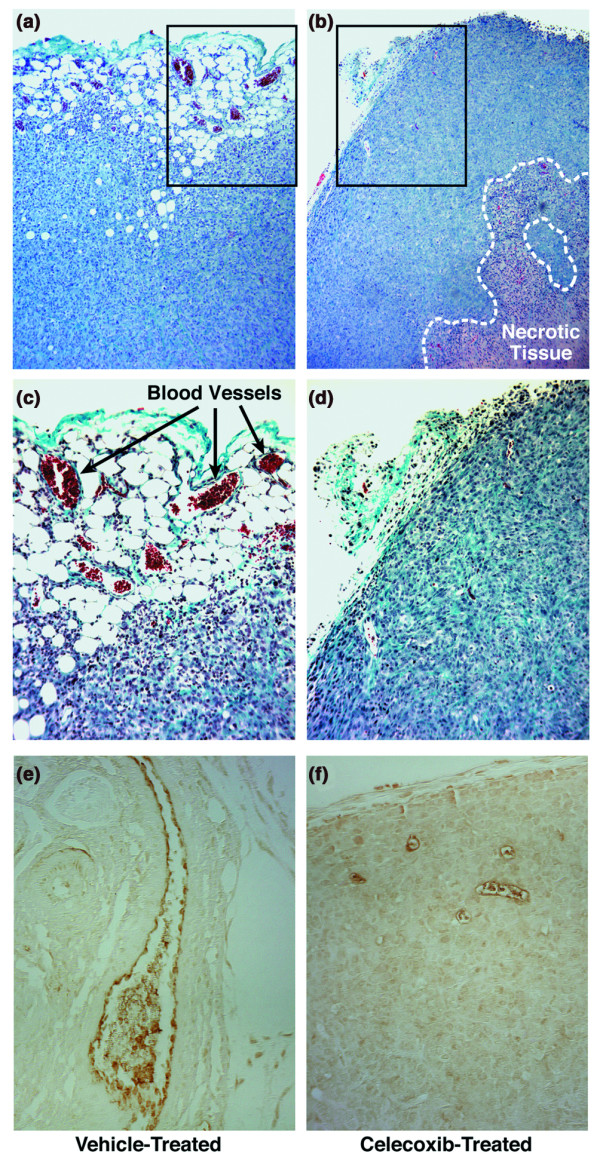
*In vivo *inhibition of angiogenesis and increase in necrosis with celecoxib treatment. Vascularity of tumor implants was histologically evaluated by Masson's trichrome and factor VIII related antigen staining. Shown is evidence of central necrosis and decreased number of blood vessels in **(b) **a section of celecoxib-treated tumors relative to **(a) **a section obtained from a vehicle-treated animal (magnification 50×). Greater magnification (100×) of **(c) **panel a and **(d) **panel b are shown in the next two panels. Arrows in panel c point to blood vessels. Endothelial cells lining the blood vessels stained positively for factor VIII related antigen and showed larger blood vessels in the **(e) **vehicle-treated than in the **(f) **celecoxib-treated samples (magnification 100×).

Vascularity of tumor implants was histologically evaluated using Masson's trichrome and factor VIII-related antigen staining. Tumors from celecoxib-treated mice showed reduced blood vessels as compared with tumors excised from vehicle-treated mice (Fig. 8). Furthermore, there was evidence of necrosis in the celecoxib-treated tumors relative to those obtained from vehicle-treated animals (Fig. 8a,b).

## Discussion

The results presented here clearly show that celecoxib strongly suppresses cell growth and proliferation in both human breast cancer cell lines (Fig. 1b). However, the mechanism of antitumor effect is dependent upon COX-2 expression and the invasive properties of the cancer cell. The highly invasive MDA-MB-231 cells undergo induction of apoptosis (Fig. 2) and the less invasive MDA-MB-468 cells undergo cell cycle arrest (Fig. 4) after treatment with celecoxib. The two cell lines exhibit different levels of COX-2 protein expression, with MDA-MB-231 cells expressing much higher levels than MDA-MB-468 cells (Fig. 1a), which directly correlated with the amount of PGE_2 _production by the cells (Table 1) and their invasive properties. Our data are in good agreement with the postulate that elevated production of COX-2-induced prostanoids is a hallmark of highly metastasizing breast cancer cells [41,42]. The two cell lines regulate COX-2 protein differently after celecoxib treatment, with downregulation of the protein observed in MDA-MB-468 cells but not in MDA-MB-231 cells (Fig. 1a). In fact there was an increase in COX-2 expression in MDA-MB-231 cells at the 60 μmol/l level of celecoxib, the mechanism for which is not known. However, one or more COX-produced products may repress COX expression in a negative feedback loop. Removal of negative feedback by celecoxib treatment would result in COX-2 induction. There are similar reports on celecoxib treatment leading to strong upregulation of COX-2 protein expression in 184htert breast cancer cells [43].

Regardless of COX-2 expression and regulation patterns, celecoxib treatment reduced PGE_2 _secretion by both cell lines (Table 1), but provision of exogenous PGE_2 _reversed celecoxib-induced growth inhibition in the MDA-MB-468 cells only, and not in the MDA-MB-231 cells (Fig. 5). This suggests that celecoxib-induced growth inhibition of the highly aggressive MDA-MB-231 cells is independent of PGE_2_. Corroborating our findings are previous reports that growth inhibition induced by COX-2 inhibitors in some carcinoma cell lines can be completely abrogated by exogenous addition of PGE_2 _[44], whereas in other studies addition of PGE_2 _had no effect [45,46]. One possible PGE_2_-independent mechanism by which celecoxib may have caused apoptosis in MDA-MB-231 cell lines could be through the accumulation of the prostaglandin precursor arachidonic acid. Arachidonic acid is known to be converted to an intermediate, apoptosis-signaling compound, namely ceramide, which causes NSAID-induced apoptosis in cancer cells [47]. This phenomenon of ceramide-induced apoptosis has been proven in a murine mammary tumor cell line treated with celecoxib [18]. Because PGE_2 _is the major prostanoid released from breast cancer cells [41], we focused our studies on PGE_2 _levels. However, a possible role of other prostanoids such as PGD_2_, PGI, PGF_2α _and thromboxane_2 _cannot be ruled out, and future studies will include analyses of other prostanoids.

Thus, we observed that the mechanisms driving celecoxib-induced growth inhibition are very diverse in the two cells lines, depending upon COX-2 expression levels, invasive properties, and dependence on PGE_2_. At the cellular level, celecoxib induced the characteristic features of apoptosis in the MDA-MB-231 cells (Fig. 2). At the molecular level, activation of protein kinase B/Akt was significantly reduced at 60 μmol/l concentration of celecoxib, with increased activation of proapoptotic protein Bax and caspases 3 and 7 (Fig. 3). These results are in agreement with those of other studies in which it was suggested that activation of effector caspases 3 and 7 and Bax proteins, downstream of phosphoinositide 3-kinase/Akt inactivation, was the mechanism of celecoxib-induced tumor cell apoptosis [22,48]. Mechanisms leading to the downregulation of Akt activation are not clear. It has been suggested that inhibition of the tumor suppressor PTEN, a phosphatase that targets phosphoinositol triphosphate, or inhibition of 3-phosphoinositide-dependent kinase 1 activity may be involved [48-50].

In contrast to MDA-MB-231 cells, growth of MDA-MB-468 cells was inhibited by induction of cell cycle arrest at the G_0_/G_1 _phase of the cell cycle (Fig. 4). Similar cell cycle arrest has been reported using a murine mammary tumor cell line derived from a spontaneously occurring tumor [18], human pancreatic cancer cell lines [51], and human ovarian cancer cell lines [52]. It is not clear from our studies that celecoxib directly affects cell cycle distribution by regulating cyclin D_1 _levels, which is one of the major cyclins known to be upregulated during cancer. Preliminary data evaluating cyclin D_1 _levels in MDA-MB-468 cells after celecoxib treatment were inconclusive (data not shown) and more thorough analysis is needed. The question remains whether COX-2 induced PGE_2 _can directly regulate cyclin D_1 _or other network of cyclins, cyclin-dependent kinases (CDKs) or CDK inhibitors. For other cell types, including colon, lung and squamous cell carcinomas, it has been reported that treatment with NSAIDs results in upregulation of CDK inhibitors that regulate accumulation of cells in G_0_/G_1 _[53-55]. In breast cancer cells, this remains to be examined.

Angiogenesis plays a crucial role in tumor development and progression. COX-2 dependent PGE_2 _production represents a likely candidate for the angiogenic response observed in several tumors, including mammary tumors [36,56-58]. To explore the role played by COX-2 inhibitors in angiogenesis, we used both *in vitro *and *in vivo *model systems. Aggressive breast epithelial cells are known to differentiate into tubules when cultured on growth factor reduced Matrigel. This phenomenon is known as vasculogenic mimicry. Its presence has been reported in inflammatory breast cancer patients and is associated with reduced 5-year survival and higher percentage of recurrence [59]. Shirakawa and coworkers [40] suggested a connection between vascular mimicry and angiogenesis, based on the existence of blood flow in the vascular channels. When plated on growth factor reduced Matrigel, human breast cancer cell lines have the unique ability to form tubular channels. We showed that the more aggressive MDA-MB-231 cells generate channels more efficiently and in higher numbers than do the less aggressive MDA-MB-468 cell line (Fig. 6a). Similarly, it was shown that highly aggressive melanoma cells, when seeded on three-dimensional matrices of collagen I, form extracellular matrix-rich patterned networks that surround clusters of tumor cells; however, under the same culture conditions, poorly aggressive melanoma cells did not form the patterned networks [38]. When treated with increasing concentrations of celecoxib (40–60 μmol/l) we observed a dose-dependent decrease in the ability of both cell lines to differentiate into channels (Fig. 6a). Our findings are in accordance with those of other reports, in which capillary-like tube formation by human umbilical vein endothelial cells cocultured with COX-2 overexpressing Caco-2 cells was inhibited by a COX-2 selective inhibitor, NS-398, in a dose-dependent manner [60].

COX-2 inhibitors have already been reported to inhibit angiogenesis, and our study shows for the first time that COX-2 regulates vascular channel formation in human breast cancer cells. The mechanism of action of celecoxib in inhibiting channel formation is not known. Our data suggest that treatment with celecoxib caused a dose-dependent downregulation of VEGF in the MDA-MB-231 cells but not in the MDA-MB-468 cells (Fig. 6b). Although additional mechanisms are involved in mediating the angiogenic effects of COX-2, our data imply that COX-2 inhibitors influence angiogenesis at least in part by decreasing the release of VEGF. It was recently reported that COX-2 induced PGE_2 _stimulated the expression of angiogenic regulatory genes, including VEGF, in mammary tumor cells isolated from COX-2 transgenic mice, and that treatment with indomethacin (a nonspecific COX inhibitor) suppressed the expression of these genes *in vitro *[36]. To confirm the *in vitro *data, the antiangiogenic effects of celecoxib were evaluated in an *in vivo *xenograft model using MDA-MB-231 cell containing Matrigel implants. Results showed that celecoxib dramatically reduced the vascularity within the tumor tissue (Fig. 8). In addition, the treatment caused increased necrosis and reduced viable tissue mass within the tumor (Figs 7 and 8). Therefore, the reduced tumor burden in the treated mice can be explained in part by the inhibition of angiogenesis and confirms our *in vitro *data. Previous studies have reported similar effects of COX-2 inhibitors in an *in vivo *angiogenesis assay using the highly metastatic murine mammary tumor cell line C3L5 [45]. Additional studies are needed to fully elucidate the complex events involved in COX-2 mediated angiogenesis in human mammary tumors.

To our knowledge, this is the first study to identify some key mechanisms of action of celecoxib *in vitro *and *in vivo *in human breast cancer cells. More cell lines must be evaluated to characterize fully the antitumor actions of celecoxib, including identification of its primary targets, the precise molecular mechanism of cell damage, and the basis for its preferential effect on tumor cells. Although COX-2 inhibitor treatment alone is unlikely to eliminate an existing tumor, it is likely that it can confer significant benefit as part of a carefully chosen regimen involving other drugs. The strategy to target multiple pathways simultaneously may be critical to improving the efficacy of therapy in the treatment of breast cancer, especially for metastatic breast cancer. Moore and coworkers [61] reported that celecoxib, in combination with 5-fluorouracil or cyclophosphamide, greatly enhanced the antitumor effects of chemotherapy in a colon cancer model. In another tumor model, COX-2 selective inhibitors showed promise in combination with radiation therapy, enhancing tumor radiation responses [62]. Celecoxib was recently shown to have chemopreventive effects against the development of chemically induced mammary tumors in the rat [12]. Finally, recent evidence that combined treatment with a nonselective NSAID and EGFR tyrosine kinase inhibitor significantly decreased polyp formation in Min APC^+/- ^mice supports the notion that combination therapy may be more effective [63]. These studies, combined with the present study and the reports of aberrant COX-2 expression in human breast cancer [9,64], suggest that selective COX-2 inhibitors have an important role to play in chemoprevention, chemo-intervention, and therapy of human breast cancer.

## Conclusion

We showed that the mechanisms driving celecoxib-induced growth inhibition of human breast cancer cells are dependent upon COX-2 expression levels, invasive properties, and dependence on PGE_2_. At the cellular level, celecoxib induced apoptosis in highly invasive cells, but it caused cell cycle arrest at the G_0_/G_1 _phase of the cell cycle without causing apoptosis in the less invasive cells. At the molecular level, pAkt was inactivated with increased activation of proapoptotic protein Bax and caspases 3 and 7. Furthermore, we showed for the first time that celecoxib inhibited microvascular channel formation in a dose-dependent manner, associated with downregulation of VEGF in the highly invasive cells. An *in vivo *xenograft model confirmed the *in vitro *data and showed dramatic reduction in tumor mass accompanied by reduced vascularity and increased necrosis within the tumor, suggesting that the reduced tumor burden in the treated mice may in part be due to reduced angiogenesis.

## Abbreviations

CDK = cyclin-dependent kinase; COX = cyclo-oxygenase; DMEM = Dulbecco's modified eagle medium; DMSO = dimethyl sulfoxide; FCS = fetal calf serum; NSAID = nonsteroidal anti-inflammatory drug; pAkt = phosphorylated Akt; PBS = phosphate-buffered saline; PG = prostaglandin; PI = propidium iodide; VEGF = vascular endothelial growth factor.

## Competing interests

The author(s) declare that they have no competing interests.

## Authors' contributions

GDB conducted the mouse studies including daily gavaging, palpating tumors and monitoring tumor growth, as well as end-point assays such as apoptosis, and caspase assays. LBP and TLT performed the western blotting and PGE_2 _assays. SJG provided expert scientific advice with regard to the MTag transgenic mice and mammary carcinogenesis. PM is the PI of the laboratory in which all experiments were conducted and is the recipient of the grant that funded the project. She was instrumental in writing the manuscript.

## Figures and Tables

**Table 1:** Celecoxib inhibited prostaglandin E_2 _secretion by breast cancer cells

Treatment	MDA-MB-231	*P *value	MDA-MB-468	*P *value
Vehicle	430.0 ± 178.2		76.6 ± 15.2	
Celecoxib 20 μmol/l	30.1 ± 2.9	<0.01	39.0 ± 16.5	0.03
Celecoxib 40 μmol/l	30.7 ± 4.7	<0.01	37.3 ± 11.0	0.02
Celecoxib 60 μmol/l	37.7 ± 13.6	<0.01	30.3 ± 13.6	0.01

Forty-eight hours post treatment, conditioned medium from vehicle and celecoxib (20–60 μmol/l) treated cells were harvested and prostaglandin (PG)E_2 _levels (pg/ml) determined by enzyme-linked immunosorbent assay. In all cell lines, PGE_2 _levels were significantly reduced at all doses of celecoxib. The experiment was repeated three times and in triplicate. *P values *represent significant differences between vehicle control and celecoxib treatment.
